# Association of War-Related and Gender-Based Violence With Mental Health States of Yazidi Women

**DOI:** 10.1001/jamanetworkopen.2020.13418

**Published:** 2020-09-18

**Authors:** Katharina Goessmann, Hawkar Ibrahim, Frank Neuner

**Affiliations:** 1 Department of Psychology, Clinical Psychology, and Psychotherapy, Bielefeld University, Bielefeld, Germany; 2 Department of Clinical Psychology, Koya University, Koya, Kurdistan Region of Iraq; 3 Vivo International, Konstanz, Germany

## Abstract

**Importance:**

Yazidi women in northern Iraq have experienced severe human rights violations through attacks by the so-called Islamic State group, with severe consequences for their health. However, no studies to date have investigated how war-related and gender-based violence, including partner violence, are associated with mental health disorders in this population.

**Objective:**

To evaluate the associations between Yazidi women’s experiences of violence (ie, war violence, partner violence, enslavement) and their mental health.

**Design, Setting, and Participants:**

This cross-sectional study of 326 women was conducted in camps for displaced persons in the Kurdistan region of Iraq between January and July 2017. Participants were married women from the Yazidi population in northern Iraq who were affected by Islamic State attacks. Participants were selected via household-randomized sampling. Data analysis was conducted from December 2018 to September 2019.

**Exposures:**

Experiences of enslavement, war-related events, and intimate partner violence were measured with event checklists.

**Main Outcomes and Measures:**

Posttraumatic stress disorder (PTSD) and depression levels were measured using culturally validated instruments.

**Results:**

A total of 326 women (mean [SD] age, 34.3 [12.9] years) participated in the study. Almost all participants reported the experience of at least 1 war-related violent event (325 [99.7%]), 54 (16.6%) reported a history of abduction and sexual slavery, and 215 (66.0%) reported the experience of at least 1 type of intimate partner violence in the past year. There were no significant differences between women who did and did not experience abduction regarding exposure to intimate partner violence. Rates of PTSD and depression symptoms were high among the whole sample, and women who experienced abduction reported significantly higher levels of psychopathology than those who did not (mean [SD] PTSD score: 61.48 [12.38] vs 47.61 [14.42]; *t*_324_ = −6.91; *P* < .001; mean [SD] depression score: 3.07 [0.68] vs 2.43 [0.68]; *t*_324_ = −6.78; *P* < .001). Multivariate hierarchical regressions revealed that psychopathology was associated with exposure to war-related events (PTSD: β = 0.29; *P* < .001; depression: β = 0.27; *P* < .001) as well as with exposure to gender-based violence in Islamic State captivity (PTSD: β = 0.19; *P* = .001; depression: β = 0.28, *P* < .001) and in their marriage (PTSD: β = 0.13; *P* = .008; depression: β = 0.18; *P* < .001).

**Conclusions and Relevance:**

In this study, interviewed Yazidi women often experienced intimate partner violence as well as war-related and gender-based violence under Islamic State attacks and enslavement, experiences that were associated with mental health impairment. The findings underline the importance of also addressing gender-based violence within health care approaches for war-affected populations.

## Key Points

**Question:**

Is gender-based violence associated with greater impairment of mental health than experiencing war-related violence alone?

**Findings:**

In this cross-sectional study among 326 displaced Yazidi women with high levels of war exposure, enslavement experience, and intimate partner violence, posttraumatic stress disorder and depression were found. Hierarchical regression analyses showed associations of war-related and gender-based violence with posttraumatic stress disorder and depression symptoms.

**Meaning:**

The findings of this study suggest that addressing gender-based violence in war-affected populations may be warranted to care for women’s mental health.

## Teaser

This cross-sectional study evaluates the associations between Yazidi women’s experiences of violence (ie, war violence, partner violence, enslavement) and their mental health.

## Introduction

Women’s health is strained in armed conflict and human emergency settings. Like all members of war-affected societies, women living in conflict zones can experience mass violence, such as massacres, bombings, and the destruction of homes as well as injuries and deaths, often followed by displacement and ongoing insecurities and poverty. However, war and its aftermath affect women in specific ways.^1,2^ Women are vulnerable to violent acts and adversities during war that are specifically related to their gender. For example, rape and sexual violence against women have reportedly been used as part of warfare strategies in conflicts across the world^3,4^ with devastating consequences.^5^ While men are usually more likely to be killed as combatants or civilians, women risk being abducted into slavery, which commonly involves torture and sexual exploitation.^6^

Women’s exposure to violence continues after the war. There is evidence that ongoing gender-based violence (GBV) is increased in regions affected by migration and displacement.^7,8^ In particular, intimate partner violence (IPV) against women is a global phenomenon that is related to sociocultural factors, such as gender inequality.^9,10^ Various studies have investigated IPV in war contexts, for example among displaced couples from Palestine, Lebanon, Uganda, Sri Lanka, Afghanistan, and Liberia^6,11,12,13,14^ as well as in camps hosting people who are affected by the ongoing war in Syria.^15^ Associations with posttraumatic stress disorder (PTSD) and depression symptoms have been found among women exposed to IPV in postcombat and displacement settings.^13^ The increased prevalence of IPV in war-affected populations^16^ may be explained at least in part by the association between trauma and aggression among war-affected men.^17^ In fact, recent research found that the men’s impaired mental health and their attitudes toward women interacted in the explanation of interpersonal violence reported by their wives.^18^

It is likely that an intertwined occurrence of war-related and gender-based forms of violence contributes to health issues in women who have been affected by wars. PTSD and depression are the most prevalent psychological disorders among forcibly displaced persons,^19,20^ and both are affected by the load of potentially traumatic experiences.^21,22^ Some studies have indicated that women are more likely to develop PTSD compared with men,^23,24^ which may be related to gender-specific risk factors that come into play both before and after exposure to traumatic war events.^25,26^ Here, we assume that gender-related stressful experiences contribute to mental health impairment among women, which may partly explain the gender difference in mental health disorders following violent conflicts.

The present study focuses on women of the Yazidi population in the Kurdistan region of Iraq (KRI), who are among those most affected by the ongoing conflicts in Iraq and neighboring countries.^27,28^ The Yazidis are a Kurdish minority, whose main distinctive feature is their orally transmitted religion, Yazidism.^29,30^ Most of the estimated 1 million Yazidis have been living in northern Iraq, in the Mosul area, and in the western areas of the KRI, which they have considered their homeland since the 12th century.^31^ Following the rise of the so-called Islamic State of Iraq and Syria (ISIS) militant group, the Yazidi population in northern Iraq experienced tremendous human rights violations. In August 2014, ISIS fighters attacked the Mount Sinjar area near Mosul, which resulted in a genocide against the Yazidi population, leaving approximately 3100 dead and approximately 6800 abducted.^32^ Women and girls were the primary targets for abduction into sex slavery and mass sexual violence.^33^ An estimated 300 000 Yazidis have resettled across the KRI, most of them in displacement camps, and thousands of others are missing.^34^ High levels of severe physical and mental health problems have been found among the displaced population.^35^ Particularly high rates of trauma exposure, PTSD, and depression are reported for women survivors of the attacks and abductions.^36,37,38,39^ The aim of this study was to determine levels of psychopathology (ie, depression and PTSD) and experiences of war-related organized violence and interpersonal violence among a sample of married Yazidi women survivors of the ISIS attacks in the KRI. In addition, the study aimed to investigate to what extent different types of war-related violence and GBV during and after the war contributed to women's psychopathology.

## Methods

### Design, Setting, and Participants

A sample of 326 Iraqi women from the Yazidi community were included in the data collection, which was carried out between January and July 2017. Participants were living in camps for internally displaced persons in the Erbil and Sulaymaniyah governorates, KRI. The sample is a subsample of a previously conducted study among Yazidi women,^36^ selected on the basis of their marital status. Sampling was performed by subdividing the camps into 6 to 7 sections and choosing households randomly by spinning a pen from the center of each subsection. The selected households were visited by trained staff to identify women eligible for participation. From each family, up to 2 women with a minimum age of 17 years were included in the study. Because we planned to include exposure to male-perpetrated IPV during the previous year in our analyses, only women who had been with a married partner within the year before assessment were included.

Data collection was carried out by 10 local female interviewers specialized in clinical psychology and experienced in work with displaced populations in the KRI. Interviewers received a 1-week theoretical and practical workshop regarding the study instruments and procedures, mental health risk management, and ethical issues. Measures taken to ensure participants’ safety during and after participation included close supervision by psychologically trained staff and the establishment of referral systems to health care organizations within and outside of the camps. Standardized interviews were conducted individually in the participants’ tents and lasted between 60 and 90 minutes. To prevent placing women reporting IPV by their husbands in danger, interviews were introduced as general surveys about health and living conditions. Informed consent was obtained from each participant in verbal form prior to assessments and was documented by the interviewers due to practical and safety reasons.^36^ All procedures of the study were approved by the ethical committees of Bielefeld University, Germany, and Koya University, KRI. This study followed the Strengthening the Reporting of Observational Studies in Epidemiology (STROBE) reporting guideline.

### Instruments

#### Sociodemographic Information

The first part of the structured interview comprised questions assessing sociodemographic characteristics of the participants. These included age, education level in years of formal education, marital status, and average income level per month as well as information on displacement (Table 1).

**Table 1.** Personal Characteristics of the 326 Participating Yazidi Women

Characteristic	Mean (SD)		
	Women who did not experience abduction (n = 272)	Women who experienced abduction (n = 54)	All women (N = 326)
Age, y	32.93 (11.31)	41.43 (17.28)	34.34 (12.86)
Formal education, y	2.21 (3.19)	1.67 (2.82)	2.12 (3.13)
Monthly income, €^a^	15.34 (70.98)	29.32 (99.94)	17.29 (75.68)
Current marital status, No. (%)			
Married	249 (91.5)	49 (90.7)	298 (91.5)
Separated or divorced	8 (2.5)	1 (1.9)	9 (2.7)
Widowed	15 (5.5)	4 (7.4)	19 (5.8)
Marriage age, y	18.06 (4.11)	17.23 (3.77)	17.93 (4.06)
Children, No.	4.11 (3.04)	4.33 (2.58)	4.15 (2.97)
Displacement experiences, No.	1.13 (0.49)	1.07 (0.26)	1.17 (0.46)
Time lived in camps, y	2.75 (0.75)	4.97 (8.14)	3.12 (3.44)
Experiences of GBV events in ISIS captivity, No.	NA	4.87 (4.01)	4.87 (4.01)
Experiences of war-related events, No.	5.45 (2.20)	9.22 (2.66)	6.08 (2.68)
Family members affected by ISIS, No.	4.94 (11.39)	16.70 (18.52)	6.89 (13.54)
Past-year experience of IPV, No. (%)	177 (65.1)	38 (70.4)	215 (66.0)
PTSD symptom level	47.61 (13.69)	61.48 (12.38)	49.91 (14.42)
Depression symptom level	2.43 (0.63)	3.07 (0.68)	2.53 (0.68)

Abbreviations: GBV, gender-based violence; IPV, intimate partner violence; ISIS, Islamic State of Iraq and Syria; NA, not applicable; PTSD, posttraumatic stress disorder.

^a^ To convert to US dollars, multiply by 1.18.

#### Mental Health

PTSD was assessed using the PTSD Checklist for the *Diagnostic and Statistical Manual of Mental Disorders* (Fifth Edition) (PCL-5).^40^ The instrument had been translated, culturally adapted, and validated among Iraqi and Syrian displaced people, including Yazidis.^41,42^ Scores on the PCL-5 scores range from 0 to 80.

Depression was assessed with the Hopkins Symptom Checklist for Depression (HSCL-D),^43^ a valid cross-cultural instrument that has been used in Kurdish and Arab populations previously.^18,41,44^ The 15 items of the HSCL-D are measured on a 4-point scale (with 1 indicating not at all and 4 indicating extremely), and depression scores, which range between 1 and 4, are obtained by dividing the sum of the item scores by the number of all items. Internal consistencies for the PCL-5 (α = .87) and the HSCL-D (α = .89) were high in this sample.

#### War and ISIS Exposure, Including Family Affectedness

**Figure 1.**
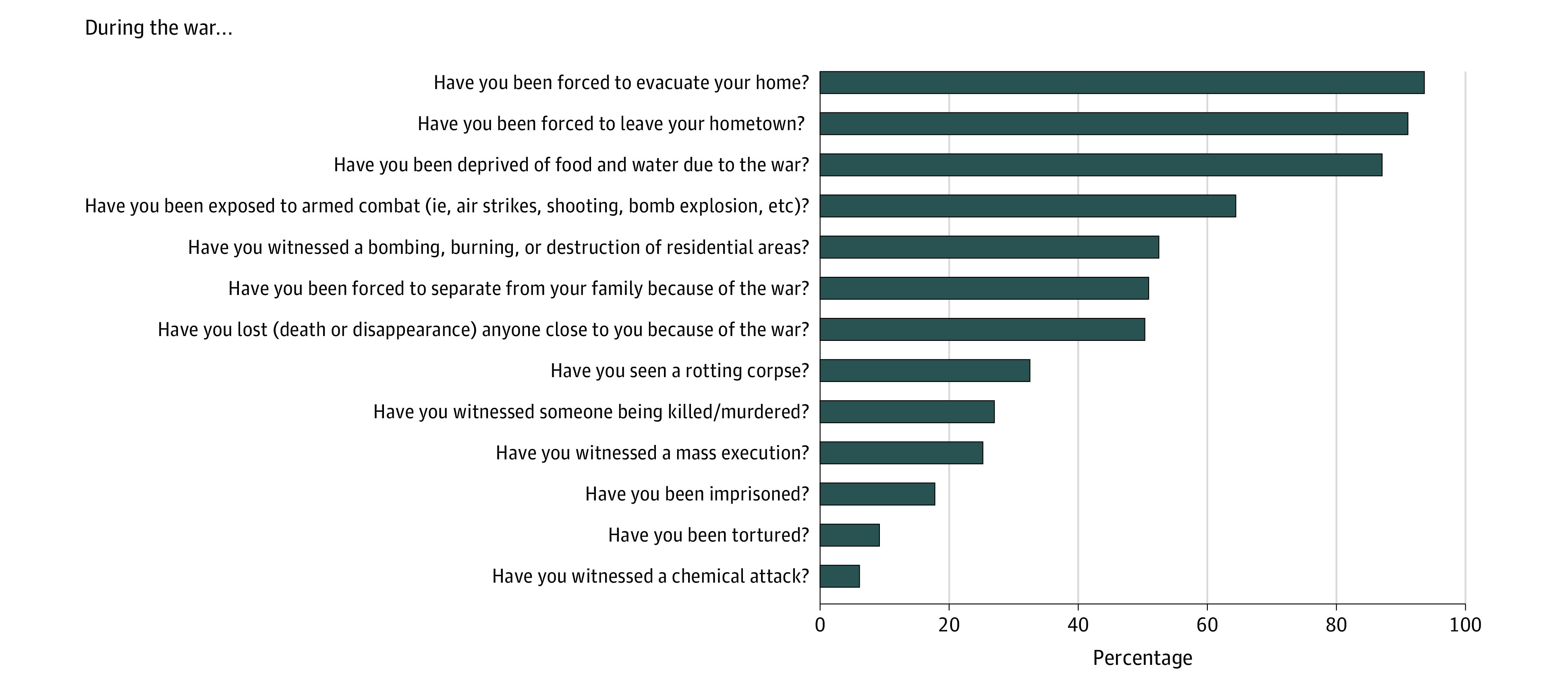
Yazidi Women Reporting Violent Experiences During War

Scales specifically developed for the context were used to assess war exposure and experiences under ISIS, including family affectedness. The war exposure scale^41^ includes 13 items covering Kurdish and Arab individuals’ experiences of war-related violence (Figure 1). A short scale of 4 items asked for the number of family members affected by ISIS attacks (ie, “How many of your family members were injured, were killed, were captured, or were disappeared by or due to ISIS?”).

#### GBV

**Figure 2.**
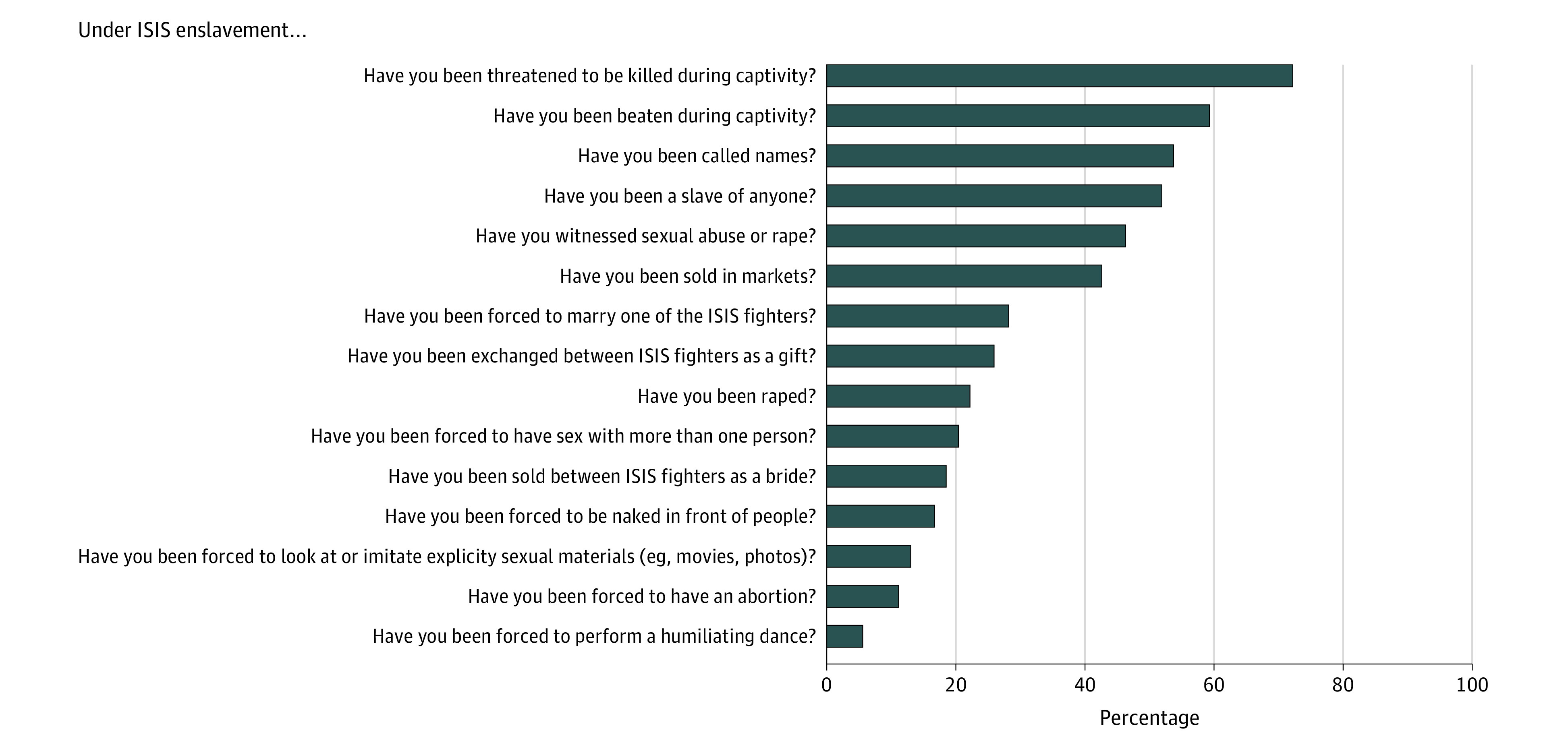
Experiences of Gender-Based Violence Among 54 Yazidi Women in Islamic State of Iraq and Syria (ISIS) Captivity

Women’s experiences of enslavement and violence under ISIS were assessed using the Enslavement Trauma Scale (ETS).^36^ The ETS is a 23-item checklist, which was developed on the basis of open discussions with ISIS enslavement survivors and local experts (psychologists, social workers, physicians, and aid workers) who work with Yazidi genocide survivors. The ETS list asks for GBV and dehumanizing treatment against women experienced during abduction, such as physical and psychological attacks, rape, forced marriage to ISIS members, objectification, public humiliation, and so on. The item list is displayed in Figure 2.

**Figure 3.**
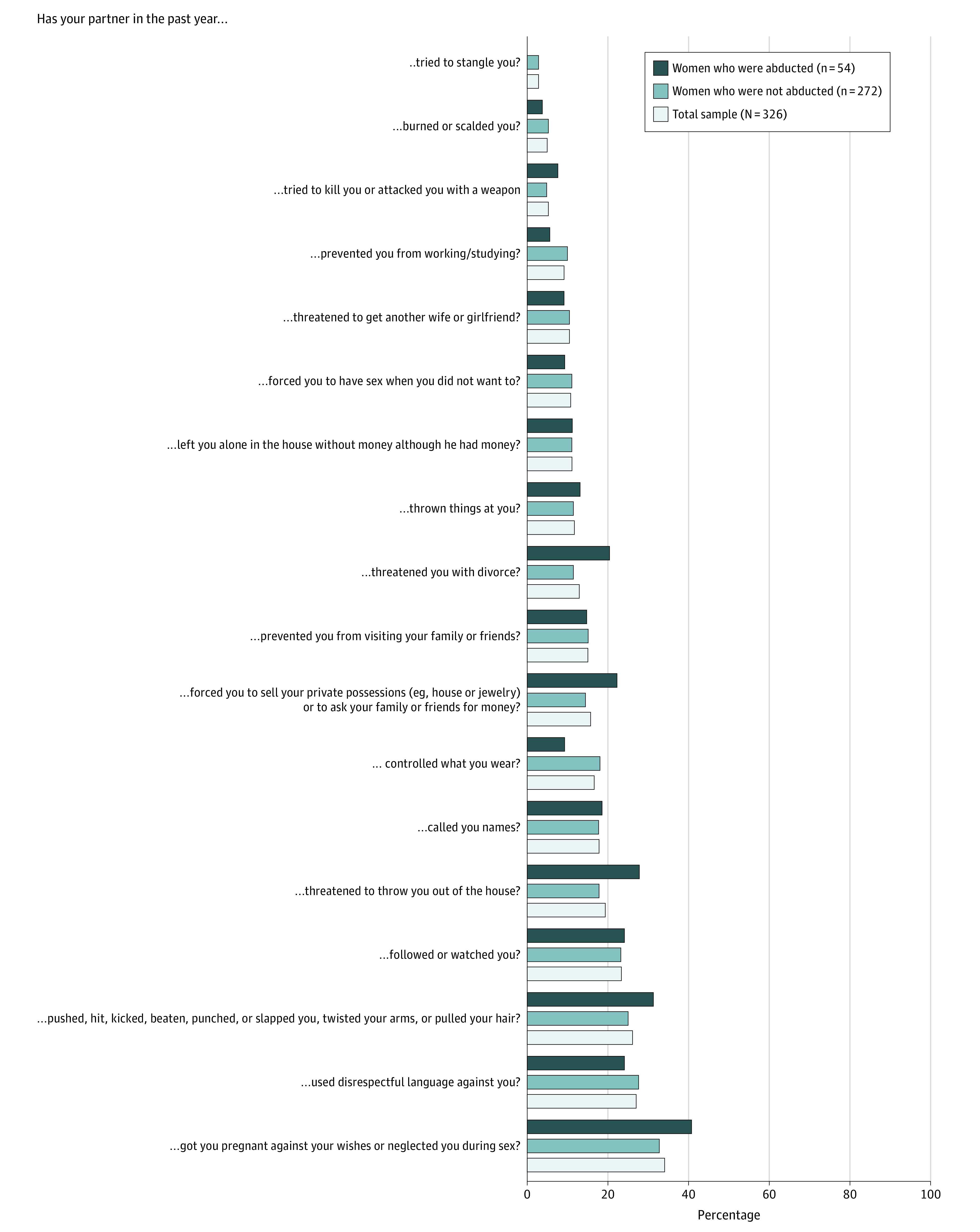
Intimate Partner Violence Acts Experienced by Yazidi Women During the Past Year

Past-year IPV was measured with an 18-item checklist covering acts of physical, sexual, psychological, and economic forms of violence against women in partnerships (Figure 3). The checklist was developed and validated on the basis of focus group discussions with displaced women from Iraq and Syria to cover IPV events relevant to women in that context (K. Goessmann, MSc, unpublished data, August 2020).

### Statistical Analysis

Descriptive analyses were used to determine levels of exposure to violence, including enslavement, war-related experiences, and IPV as well as psychopathology. Group differences between women who did and did not experience abduction were analyzed using the *t* test. To investigate how different forms of violence experienced by women were associated with their levels of PTSD and depression symptoms, 2 multivariable hierarchical regression analyses were calculated; the sum scores of PTSD and depression were introduced as outcome variables. In both regression models, women’s age, education, and income levels were entered in the first step to control for sociodemographic influences on mental health. In the second step, women’s and their families’ war-related violence exposure were entered as independent variables. In the last step, variables of GBV (ie, experiences of GBV in ISIS captivity and IPV) were entered. All tests were 2-sided, with a significance level of *P* < .05. Analyses were performed using SPSS statistical software version 25 (IBM Corp). Data analysis was conducted from December 2018 to September 2019.

## Results

### Descriptive Results on Violence Exposure and Psychopathology Among Yazidi Women

The participating 326 women had a mean (SD) age of 34.3 (12.9) years. Most were married (298 [91.5%]), others were divorced (9 [2.7%]) or widowed (19 [5.8%]); however, all had been with a married partner within the year prior to assessments.

Analyses showed that the interviewed women were highly affected by war-related violence as well as gender-based violence. Overall, 54 (16.6%) reported that they had been abducted by ISIS. High levels of exposure of family members were also reported; a mean (SD) of 6.89 (13.54) family members had been killed, kidnapped, or injured by ISIS. Almost all participating women (325 [99.7%]) reported that they had experienced at least 1 war-related violent event, and those who had been abducted by ISIS reported significantly more mean (SD) events than those who had not been abducted (9.22 [2.66] events vs 5.45 [2.20] events; *t*_324_ = −11.08; *P* < .001). Figure 1 displays percentages of women reporting the experience of different violent events during war. The 3 most frequently experienced war events were being forced to evacuate one’s home (305 women [93.6%]), being forced to leave one’s hometown (297 [91.1%]), and being deprived of food or water (284 [87.1%]).

Among those abducted by ISIS, 50 (92.6%) experienced a mean (SD) of 4.87 (4.07) different acts of physically and sexually violent and humiliating acts in captivity. Among the most common forms of GBV in captivity were sex slavery (reported by 28 [51.9%]) and being forced to convert to Islam (43 [79.6%]). Figure 2 provides more details regarding GBV experienced by women in captivity.

IPV was also highly prevalent in this sample (Figure 3). Two-thirds (215 [66.0%]) of all interviewed women reported having experienced at least 1 type of IPV from their husband within the past year. The most common acts were verbal attacks (reported by 88 [27.0%]), physical attacks (85 [26.1%]), threats by the husband to get another wife (76 [23.3%]), and sexual violence in terms of disrespecting the women’s sexual and reproductive autonomy, including forced pregnancies (111 [34.0%]). There were no significant differences between women who had and had not experienced abduction regarding the number of experienced IPV events (*t*_324_ = −0.42; *P* = .68).

Regarding the mental health of the participants, both depression and PTSD symptom levels were high, especially among women who had been abducted by ISIS. The mean symptom levels of the whole sample were 49.91 (SD, 14.42; range, 8-80) on the PCL-5 and 2.53 (SD, 0.68; range, 1.13-4.00) on the HSCL-D. Women who had been abducted by ISIS experienced significantly higher levels of psychopathology both in terms of PTSD (mean [SD] score, 61.48 [12.38] vs 47.61 [14.42]; *t*_324_ = −6.91; *P* < .001) and depression (mean [SD] scores: 3.07 [0.68] vs 2.43 [0.68]; *t*_324_ = −6.78; *P* < .001). A summary of sociodemographic, psychological, and violence-related characteristics of the sample is displayed in Table 1.

### Associations of Violent Experiences With PTSD and Depression

Using multiple hierarchical regression analyses, associations of PTSD and depression scores with different forms of violence exposure were assessed. As shown in Table 2, war-related violence as well as GBV were associated with the women’s mental health issues, both in terms of PTSD and depression (war-related events, PTSD: β = 0.29; *P* < .001; war-related events, depression: β = 0.27; *P* < .001; GBV in ISIS captivity, PTSD: β = 0.19; *P* = .001; GBV in ISIS captivity, depression: β = 0.28, *P* < .001; IPV, PTSD: β = 0.13; *P* = .008; IPV, depression: β = 0.18; *P* < .001). Symptom levels were most strongly associated with women’s experiences of war-related violence (step 2), but IPV and enslavement-related GBV exposure were associated with a worse outcome over and above the association of mental health with war exposure (step 3). GBV (both in ISIS captivity and as IPV, step 3) had a particularly high correlation with depression symptomatology, given that entering the GBV variables in the regression explained an additional 8% of variance compared with 4% in the case of PTSD. In total, the models explained 25% of variance of PTSD and 29% of variance for depression symptoms.

**Table 2.** Multiple Hierarchical Regression Analysis of Mental Health Issues Among 326 Yazidi Women

Factor	PTSD				Depression			
	B (SE)	β	*t*	*P* value	B (SE)	β	*t*	*P* value
Step 1								
Age	0.10 (0.06)	0.09	1.65	.10	0.12 (0.04)	0.15	2.68	.008
Education	0.03 (0.26)	0.01	0.12	.90	0.05 (0.18)	0.02	0.28	.78
Income	0.00 (0.00)	0.14	2.51	.01	0.00 (0.00)	0.10	1.84	.07
Step 2								
No. of family members affected by ISIS	0.23 (0.06)	0.22	3.97	<.001	0.17 (0.04)	0.23	4.18	<.001
War-related violence	1.72 (0.31)	0.30	5.48	<.001	1.15 (0.22)	0.29	5.18	<.001
Step 3^a^								
Gender-based violence in ISIS captivity	1.12 (0.35)	0.19	3.22	.001	1.13 (0.24)	0.27	4.73	<.001
Intimate partner violence	0.52 (0.19)	0.13	2.67	.008	0.49 (0.13)	0.17	3.64	<.001

Abbreviations: ISIS, Islamic State of Iraq and Syria; PTSD, posttraumatic stress disorder.

^a^ Variance explained by full PTSD model: *R*^2^ = .25; F_7, 318_ = 14.7; *P* < .001. Variance explained by full depression model: *R*^2^ = .28; F_7, 318_ = 17.8; *P* < .001.

## Discussion

Violence, war, and forced displacement have negative consequences on individuals’ physical and mental health, and women often experience multiple forms of violence during war and political conflict, creating multilayered burdens. This study showed that Yazidi women who survived the ISIS genocide in 2014 have experienced high levels of war-related violence as well as organized and interpersonal GBV, and most experienced PTSD and depression symptoms. Psychopathology was alarmingly high across the whole sample. The levels were highest among those who had been abducted by ISIS, reflecting findings from other contexts regarding the lasting adverse effects of the atrocities experienced in captivity by abducted individuals.^45,46^

Women survivors of ISIS abduction reported more war-related violent events than survivors who had not been abducted, but overall levels of IPV exposure were similarly high among women who did and did not experience abduction in this sample. This indicates that IPV is a common issue among displaced Yazidi couples, irrespective of the times of separation. The most common forms of IPV reported included threats of remarrying and reproductive coercion, which indicates that control and oppression are common features in the women’s everyday lives. It is likely that social norms around the subordination of women play a significant role in the continuation of violence experienced by women in postconflict settings, given that gendered social structures tend to be reinforced by conflict.^47,48^ The myriad strains affecting displaced Yazidi women were further highlighted by the results of the multiple regression analyses, which indicated that war-related violence as well as GBV contributed to high levels of psychopathology. Of all associated factors, war-related events had the highest correlations with PTSD and depression symptoms, but additional exposure to GBV both within partnerships and in captivity significantly added to the explanation of variance of mental health. This finding sheds light on the ongoing experiences of war-affected Yazidi women in Iraq by illuminating the interplay of various sources of violence embedded in unstable contexts.

The adverse health consequences of violence against women in general are well known,^49^ and detrimental health states have previously been documented for Yazidi women survivors of ISIS enslavement.^36,38,50^ Our results add to this evidence by indicating that GBV may affect women’s health above and beyond the associations of intense war violence experiences. The negative mental health effects of IPV have been shown for women across the world,^51^ including women living in postconflict settings.^13,52^ Our study extends these findings by documenting the associations of GBV with mental health beyond other types of violence exposure in postconflict settings.

On a broader note, despite limited generalizability of the findings due to the sample’s ethnic specificity, our findings underline the multidimensionality and complexity of women’s experiences in war.^47^ It is plausible to assume that attacks against women targeted the Yazidi community as a whole, with the deliberate aim to destabilize the Yazidis’ social structures.^53,54^ In fact, women who had been abducted and enslaved by ISIS risk being isolated from the community due to Yazidis’ religious traditions proscribing intimate encounters with individuals outside of the community.^55^ As our previous research among Yazidi women showed,^36^ feelings of stigmatization by their community played a role in the women’s violence-associated psychopathology. In light of the alarmingly high rates of PTSD and depression and associated violence experiences, it becomes evident that mental health services for Yazidi women are sorely needed. Such services should target social as well as psychological factors. Beyond providing evidence-based approaches to treat trauma and depression, efforts should be undertaken to increase survivors’ acceptance in their communities and to reduce ongoing IPV.

### Strengths and Limitations

This study has strengths and limitations. To our knowledge, this study is the first to reveal combined associations of several types of violence with the highly strained mental health of Yazidi women living in displacement camps in northern Iraq. However, interaction and mediation effects of different forms of violence were not analyzed here, and future studies should look more into possible interplays as well as into other factors not assessed in this study. Such factors might include camp and migration factors as well as other forms of family or community violence, which seem especially relevant in highly community-oriented societies like that of the Yazidis. Also, the explanatory potential of our study is limited due to its cross-sectional design, not allowing temporal or causal interpretations. Future studies should use longitudinal designs to determine the temporal sequence of violent experiences and resulting effects.

## Conclusions

Women are among those experiencing the greatest levels of trauma during violent conflicts. As the findings of this study among Yazidi women survivors of the ISIS genocide demonstrate, more needs to be done to address the complex interplays of violence experienced by women during war and after, such as addressing mental health needs and preventing ongoing GBV violence like IPV.
